# Epistaxis: A Rare Presentation of Sickle Cell Intrahepatic Cholestasis

**DOI:** 10.1155/crh/2660044

**Published:** 2025-08-24

**Authors:** Boraan Abdulkarim, Hannah Cushen, James Grace, Erica Levine

**Affiliations:** ^1^Department of Pediatrics, Loyola University Medical Center, Maywood, Illinois, USA; ^2^Department of Pediatrics, University of Minnesota Medical Center, Minneapolis, Minnesota, USA; ^3^Department of Medicine, University of Minnesota Medical Center, Minneapolis, Minnesota, USA

**Keywords:** bilirubinemia, hepatology, liver transplant, SCD, SCH, SCIC, sickle cell disease, sickle cell hepatopathy

## Abstract

Sickle cell hepatopathy (SCH) is an umbrella term relating to liver disease in sickle cell disease (SCD). This term ranges from common etiologies such as cholelithiasis to disease-specific causes such as sickle cell intrahepatic cholestasis (SCIC), a rare but significant complication of SCD capable of progressing to liver failure and consideration of transplantation. We report the case of a 24-year-old male with SCD who presented with jaundice, encephalopathy, uncontrollable epistaxis, and pseudohematemesis and was found to have hyperbilirubinemia, coagulopathy, portal hypertension, and acute kidney injury (AKI). This presentation was concerning for SCIC. Initial management included transfusions and a trial of apheresis. Liver biopsy revealed sinusoidal red cell sickling, fibrosis, and ductopenia, consistent with findings of SCIC. Due to ongoing complications, recurrent admissions, and symptomatic coagulopathy, the patient underwent liver transplantation which was complicated by perihepatic hematoma and stroke, necessitating extensive rehabilitation. This case emphasizes the importance of early diagnostic workup and prompt, multidisciplinary management of SCIC to mitigate risks of liver failure and need for transplant.

**Summary**

• This case report aims to contribute to the growing understanding of sickle cell hepatopathy (SCH) by emphasizing diagnostic challenges and multidisciplinary management strategies.
• These insights underscore the importance of addressing research gaps in the pathogenesis and treatment of SCD-related liver and vascular complications.
• Data sources were obtained from the University of Minnesota Medical Center (UMMC).
• Data were extracted from clinical records and a literature review.

## 1. Introduction

Sickle cell disease (SCD), marked by two copies of hemoglobin S gene, is a chronic disease that causes fragility of hemoglobin and consequently the erythrocyte. Fragile misshapen, or sickled, red blood cells can accumulate in the vasculature, causing pain crises pathognomonic of the disease. The term sickle cell hepatopathy (SCH) encompasses liver disease found within patients with SCD. This dysfunction can range from mild jaundice to fulminant hepatic failure with complex coagulopathies. Sickle cell intrahepatic cholestasis (SCIC) is a rare but significant etiology of SCH caused by sickling in the hepatic sinusoids. This case highlights the complexities of determining the etiology of SCH in a patient with severe hyperbilirubinemia, coagulopathy, and portal hypertension.

Our patient presented with jaundice, hyperbilirubinemia, and uncontrolled epistaxis which were initially linked to his SCD status. However, the etiology of his coagulopathy and ultimately his SCH was unknown initially with common causes such as extrahepatic obstruction needing to be ruled out. Patients with SCH must be treated as a high bleeding risk, including using minimally invasive biopsy techniques especially when concurrent portal hypertension is present. These patients also benefit from collaboration between hematology, hepatology, and transplant services to optimize outcomes in future cases, as ours did.

We put forward this case which sets precedent for severe epistaxis as a presenting sign of coagulopathy resulting from liver failure due to SCIC. This case report aims to contribute to the growing understanding of SCH and SCIC by emphasizing diagnostic challenges and multidisciplinary management strategies. These insights underscore the importance of addressing research gaps in the pathogenesis and treatment of SCD-related liver and vascular complications.

## 2. Case Presentation

A 24-year-old man with SCD and prior cholecystectomy presented with severe epistaxis, pseudohematemesis, back pain, acute kidney injury (AKI), jaundice, and encephalopathy. Labs showed direct hyperbilirubinemia (38 mg/dL), hemoglobin of 4.3 g/dL (baseline 7-8 g/dL), platelets of 88,000/mcL, AST of 184 U/L, and INR of 2.5.

Imaging ruled out biliary obstruction and suggested diffuse parenchymal disease. The patient's AST was higher than expected for hemolysis but PEth was normal, indicating SCH with potential SCIC as the etiology. His thrombocytopenia and coagulopathy were attributed to worsening liver function and sequestration.

Fresh frozen plasma (FFP) and packed red blood cells (pRBCs) were transfused, decreasing INR to 1.55. No platelet transfusion was administered; however, his platelets increased to 100,000/mcL. Despite a trial round of apheresis during his admission, his bilirubin remained elevated at 32.

**Figure 1:**
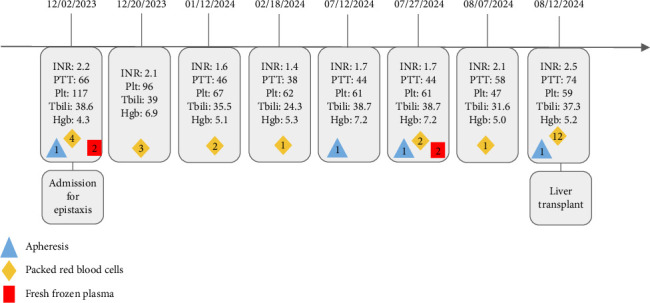
Each admission from epistaxis presentation through liver transplantation is listed with admission laboratory levels and if the patient received any transfusions during that admission. The number of units or occurrences is enumerated within the shape from the legend.

Due to bleeding risk, liver biopsy was deferred and transplant evaluation was begun. He was discharged on lactulose and ursodiol but within months was readmitted for recurrent epistaxis and acute anemia with persistent hyperbilirubinemia and coagulopathy. Throughout numerous admissions, he underwent multiple transfusions and apheresis treatments, as outlined in Figure 1.

Months later, liver biopsy was pursued which showed hepatic congestion due to sinusoidal red cell sickling associated with fibrosis and ductopenia consistent with SCIC as the etiology of his SCH. He continued to have multiple admissions for liver disease over the course of the next year. Workup for transplantation continued including consideration of blood product needs, a plan for an exchange transfusion prior to transplantation, and close follow-up with his multidisciplinary teams. Ultimately, he received a liver transplant which required an exchange transfusion the day prior as well as multiple transfusions of blood products along with tranexamic acid 1 g every 8 h peri-op. Unfortunately, his transplant was complicated by perihepatic hematoma and stroke. He has had multiple admissions for complications since but continues to work on rehabilitation posttransplant.

## 3. Discussion

Direct hyperbilirubinemia in sickle cell patients warrants a broad differential for all potential etiologies of SCH in order to provide quick diagnosis and management. Best practice for his SCH-related hyperbilirubinemia was treatment with apheresis and liver transplantation; however, his symptomatic coagulopathy made apheresis high risk and the decision was made to stop apheresis after one cycle. This case underscores the importance of thorough workup for sickle cell patients, who are at risk of anchoring bias [1], as well as maintaining a broad differential when patients present with SCH.

The exact pathophysiology of SCIC is poorly understood in the literature—cholestatic jaundice in patients with SCD was historically determined to be sickle cell cholangiopathy [2]. Intravascular sickling can congest in portals critical to blood flow, such as in the hepatic portal system, as was present on biopsy in our patient. The consensus pathophysiological explanation for SCIC is sinusoidal vaso-occlusion with hepatic ischemia and canalicular cholestasis, potentially exacerbated by an inflammatory response [3].

SCIC treatment options, both pharmaceutical and nonpharmaceutical, are lacking in research, specifically in the way of randomized controlled trials [4]. The most established standard of treatment for SCIC and resulting hyperbilirubinemia is plasmapheresis [5–7]; however, as SCD patients progress through chronic congestion and into end-stage liver disease (ESLD), liver transplantation is increasingly recognized as a treatment for ESLD in SCIC patients. The risk of posttransplant complications remains an area of concern. A 2006 case study and literature review concluded that while liver transplantation does not alter the underlying pathophysiology of sickle cell–related vascular thrombosis, it can provide life-saving support in cases of liver failure [8]. Recent studies show promise for improvement with liver transplantation. Survival rates were reported to improve in transplanted patients with acute liver failure (ALF) due to SCH [9]. However, liver transplantation remains associated with marked early postoperative morbidity and mortality [9]. 1-year survival of liver transplant in patients with SCD and ALF is approximately 88.5% [1, 8], although social determinants of health affecting this patient population are yet to be removed as confounding factors [1].

Phototherapy was considered due to our patient's persistent hyperbilirubinemia. Preliminary research shows some promise that light therapy may provide alternative treatment avenues for adults [10], and specifically SCIC patients [11, 12]. However, given our patient demonstrated conjugated bilirubinemia, the pathophysiology of light therapy favoring elimination of unconjugated bilirubin made this an unfavorable pursuit. While this therapy may hold future promise for patients such as this one, due to lack of evidentiary support, traditional apheresis was prioritized after discussion with hematology and gastroenterology colleagues.

Although epistaxis is a rare presentation of SCH in the west, epistaxis as a presentation in patients with SCD worldwide is common and recognized in African and tropical regions of the world. Studies have found higher rates of epistaxis in African and Indian SCD populations compared to controls, with severe cases requiring transfusions for resolution [13, 14, 15]. This may be related to a genetic link caused by haplotype [16]. The pathophysiologic connection between SCD sequelae and epistaxis, such as vascular fragility and coagulopathy, remains underexplored, making our case particularly relevant, as the epistaxis for our case seemed more related to his severe coagulopathy rather than related to nasal vascular fragility. The rarity of our patient's presentation in the west contrasted against the literature's documentation of global patterning of this presenting symptom also underscores increased need for inquiry into the genotypic, social, and environmental modulators of SCH progression and pathophysiology.

Our patient's presentation, characterized by refractory epistaxis, severe hyperbilirubinemia, and intrahepatic cholestasis, joins many themes in the literature regarding SCH. Unlike previous cases with more chronic progression between initial hyperbilirubinemia [3] and symptomatic coagulopathy [17], ours presented with the two concurrently. Unlike previous studies that segregated epistaxis as a symptom more common in SCD patients in the tropics and Africa [13, 15, 16], our patient has always lived in the United States. Our patient progressed in liver disease despite plasmapheresis, received a liver transplant, and is suffering postoperative morbidity as is documented in the literature [1, 8, 9].

This case contributes to the growing understanding of SCH by presenting a rare instance of SCIC and epistaxis presenting simultaneously in a U.S. patient. It underscores the importance of comprehensive diagnostic workups, early intervention, multidisciplinary management, and the exploration of novel therapeutic strategies, such as phototherapy and exchange transfusion, in the management of sickle cell–related complications.

## 4. Conclusions

This case purveyed a rare instance in which SCIC led to portal hypertension and ultimately resulted in liver failure and a severe coagulopathy that presented as uncontrollable epistaxis, the first presentation of which to be put forth in the United States. His severe epistaxis expanded the differential beyond sickle cell crisis leading to the diagnostic workup and prompt treatment which are essential in attempting to prevent liver failure and reducing transplant-related risks. Swift involvement of transplant and hepatology teams expedited his candidacy for liver transplant when his liver disease progressed. Keeping SCIC on the differential is essential for any patient presenting with SCH and warrants discussion of therapies such as plasmapheresis and liver transplantation.

## Data Availability

Data sharing is not applicable to this article as no new data were created or analyzed in this study. Information from this manuscript is available from the corresponding author upon reasonable request.
